# Isoniazid-resistant tuberculosis: A problem we can no longer ignore

**DOI:** 10.1371/journal.pmed.1003023

**Published:** 2020-01-21

**Authors:** Giorgia Sulis, Madhukar Pai

**Affiliations:** 1 Department of Epidemiology, Biostatistics and Occupational Health, McGill University, Montreal, Quebec, Canada; 2 McGill International TB Centre, McGill University, Montreal, Quebec, Canada; 3 Manipal McGill Program for Infectious Diseases, Manipal Centre for Infectious Diseases, Manipal Academy of Higher Education, Manipal, Karnataka, India

## Abstract

Giorgia Sulis and Madhukar Pai discuss the global distribution, and approaches to diagnosis and treatment, of isoniazid-resistant tuberculosis,

For decades, people working in tuberculosis (TB) knew that monoresistance to isoniazid (INH) was common. INH has been in clinical use since the 1950s, and drug resistance was expected because its use became widespread. But this knowledge did not necessarily lead to testing for INH-resistant, rifampicin-susceptible TB (Hr-TB) or to the use of special drug regimens for this form of TB. Indeed, for decades, no drug-susceptibility testing (DST) for any drug was done unless patients failed first-line therapy or had risk factors for drug-resistant TB (DR-TB). Simply put, we chose to ignore the problem.

When the TB world woke up to the need for universal DST and included it as a key goal in the End TB Strategy released in 2015, the focus became rapid testing for rifampicin resistance (RR) as a means of achieving universal DST. Novel technologies such as Xpert MTB/RIF (Cepheid, Sunnyvale, CA, USA) were rolled out in 2010, but the technology did not include INH-resistance testing [1]. Even today, access to any DST remains low, and when performed, DST is often limited to RR [2].

In 2020, we can no longer hide from this worrisome problem because Hr-TB is much more common than RR and could seriously jeopardize progress in the fight against TB. This is confirmed by an analysis of aggregated drug resistance data from 2003 to 2017 across 156 countries presented in the accompanying research study by Anna Dean and colleagues in *PLOS Medicine*, showing that—on average—7.4% (95% CI 6.5–8.4) of new cases and 11.4% (9.4–13.4) of previously treated patients have Hr-TB [3]. The overall prevalence of INH resistance (with or without concomitant RR) ranged between 10.7% (9.6–11.9) and 27.2% (24.6–29.9) depending on the treatment history and reached even more alarming levels in certain countries, particularly in the European and Western Pacific regions.

The analysis by Dean and colleagues highlights major flaws in national surveillance systems, which go hand in hand with limited laboratory capacity. The small sample sizes available from some countries make national prevalence estimates imprecise. Furthermore, the diversity of detection methods employed across settings along with the widespread lack of quality control underscores the need for improved surveillance by countries.

From a clinical standpoint, if INH resistance is not detected, new patients are managed as if they had pansusceptible TB, with a substantially increased risk of treatment failure or relapse and a greater propensity to acquire further resistance [4]. Yet, most research and policy efforts so far have been focused solely on RR as a proxy for multidrug-resistant (MDR)-TB. This means that hundreds of thousands of patients with Hr-TB are staying in the shadows, not receiving appropriate care, and all too often ending up developing MDR-TB.

Thankfully, some progress has been made in the last couple of years. In 2018, the World Health Organization (WHO) issued new treatment recommendations for Hr-TB, replacing the previous 9-month course of rifampicin, pyrazinamide, and ethambutol (RZE) with a 6-month regimen based on levofloxacin plus RZE [5,6]. This recommendation is reflected in the updated guideline for DR-TB jointly released in 2019 by the American Thoracic Society, Centers for Disease Control and Prevention, European Respiratory Society, and Infectious Disease Society of America, which also suggests reducing the duration of pyrazinamide to 2 months in case of noncavitary and lower-burden disease or if a high risk of pyrazinamide-induced toxic effects are anticipated [7].

However, high-TB-burden countries will struggle to routinely implement these new guidelines for Hr-TB because easy access to INH-resistance testing is a challenge. Although there are WHO-endorsed technologies (such as line-probe assays and liquid cultures) that can detect INH resistance, these tools are limited to centralized or reference laboratories [8]. Also, even in settings where the prevalence of Hr-TB is the highest, starting a quinolone-containing regimen empirically requires caution, in spite of the low levels of resistance to levofloxacin and/or pyrazinamide, as documented by a recent surveillance project [9].

The year 2020 might bring some hope, as the Xpert MTB/XDR cartridge (Cepheid, Sunnyvale, CA, USA) is expected to be released and will include resistance testing for INH, fluoroquinolones, and second-line injectables [10]. The TB diagnostics pipeline also includes several next-generation, high-throughput molecular tests that are able to simultaneously detect rifampicin and INH resistance in centralized laboratories [11,12].

Whole-genome sequencing (WGS) data continue to shed light on the wide range of mutations associated with drug resistance, thus not only improving our understanding of transmission dynamics but also helping to refine therapeutic choices for the individual patient [13]. Importantly, a deeper examination of the genotypic diversity of INH resistance would be of great benefit to inform treatment guidelines that are currently based on low-quality evidence [6]. At present, nationwide scale-up of WGS for routine TB diagnosis may not seem within reach in most high-TB-burden countries owing to cost and infrastructure requirements. However, this technology is becoming cheaper and easier and offers an incredible opportunity to generate better quality information on INH resistance, thanks to its ability to detect clinically relevant mutations that are not captured by conventional rapid tests, which usually target only the most common *katG* and *inhA* mutations [13,14]. Recently, Unitaid and the Foundation for Innovative New Diagnostics (FIND) have partnered to launch the Seq&Treat project that will pilot next-generation genome sequencing, an innovation that will enable fast, accurate diagnosis of DR-TB [15].

Beyond technologies and guidelines, we need to acknowledge the human impact of DR-TB, which imposes a tremendous physical, mental, financial, and social burden on patients. In addition to the 3 million TB patients who fail to get diagnosed or notified [11], there are large numbers of people with Hr-TB who are misdiagnosed and consequently mismanaged. Therefore, access to quality TB care is a human rights issue. In 2020, quality TB care must include universal DST—not just RR testing—for all individuals with TB, followed by individualized therapy, based on DST results.

## Abbreviations

DST: drug-susceptibility testing
DR-TB: drug-resistant tuberculosis
FIND: Foundation for Innovative New Diagnostics
INH: isoniazid
Hr-TB: isoniazid-resistant, rifampicin-susceptible tuberculosis
MDR-TB: multidrug-resistant tuberculosis
RR: rifampicin resistance
RZE: rifampicin, pyrazinamide, and ethambutol
TB: tuberculosis
WGS: whole-genome sequencing
WHO: World Health Organization
